# Design of a Wireless Ultraviolet Germicidal Irradiation System and Validation of Germicidal Potential Against Biofilm-Forming Bacteria and Fungi

**DOI:** 10.3390/antibiotics15050507

**Published:** 2026-05-18

**Authors:** Bindu Sadanandan, Shyam Sunder, Vaniyamparambath Vijayalakshmi, Priya Ashrit, Kavyasree Marabanahalli Yogendraiah, Kalidas Shetty

**Affiliations:** 1 Department of Biotechnology, M S Ramaiah Institute of Technology, Bengaluru 560054, Karnataka, India; v.viju114@gmail.com (V.V.); priya.ashrit@ndsu.edu (P.A.); kavya1999my@gmail.com (K.M.Y.); 2 Product Development and Innovation Center, Bharat Electronics Limited, Bengaluru 560013, Karnataka, India; daramss@bel.co.in; 3 Department of Plant Pathology, Microbiology and Biotechnology, North Dakota State University, Fargo, ND 58102, USA; kalidas.shetty@ndsu.edu

**Keywords:** UVGI, UVC, sterilization, biofilm, *Bacillus subtilis*, *Escherichia coli*, *Candida albicans*

## Abstract

**Background:** A compact, in-house-developed ultraviolet germicidal irradiation (UVGI) system using eight 36 W Philips low-pressure mercury UV-C lamps with a peak emission at 253.7 nm was developed for effective sterilization of bacteria and fungi using a wireless mode of operation. **Methods:** Under controlled laboratory conditions, the system was tested against representative biofilm-forming microorganisms, including *Bacillus subtilis*, *Escherichia coli* K12, and a multidrug-resistant *Candida albicans* M-207 isolate. Microbial viability was assessed using colony-forming unit (CFU) enumeration and 3-(4,5-dimethylthiazol-2-yl)-2,5-diphenyltetrazolium bromide (MTT) assay, with structural changes analyzed by scanning electron microscopy (SEM). Cultures were exposed to 253.7 nm UV-C radiation at distances of 1–5 m for 15–90 min. **Results:** UV-C exposure resulted in time- and distance-dependent reductions in viable counts for all tested organisms, as determined by CFU analysis. At 1 m and 15 min exposure, viable counts for all tested organisms were reduced below the limit of detection (LOD) of the CFU assay, indicating substantial microbial inactivation under the tested conditions. Reduced efficacy was observed at increased distances (3 m and 5 m), with log_10_ reductions varying depending on organism and exposure conditions. Residual metabolic activity detected by the MTT assay suggests the presence of non-proliferating or damaged cells, consistent with the different endpoints measured by the two assays. The SEM analysis further revealed disruption of biofilm architecture and reduction in cell density with increasing UV dose. **Conclusions:** The UVGI system demonstrated dose-dependent inactivation of biofilm-forming microorganisms under controlled conditions, supporting its proof-of-concept efficacy. Further studies are required to evaluate performance under real-world conditions.

## 1. Introduction

Microorganisms have been studied in diverse public areas such as telephone booths, toilets, schools, hospitals, and common transportation points [1,2]. Healthcare environments are recognized as high-risk settings for microbial contamination due to the continuous introduction and circulation of pathogens, while indoor environments and HVAC systems further contribute as important reservoirs and dissemination pathways for bacteria and fungi [3,4]. In the hospital environment, microorganisms, especially nosocomial pathogens, cause hospital-acquired infections, such as central line-associated blood stream infection (CLABSI), catheter-associated urinary tract infection (CAUTI), ventilator-associated pneumonia (VAP), and surgical site infection (SSI), which are of global concern [5]. The most common nosocomial pathogen is *Escherichia coli*, which is a common cause of urinary tract infections. Other pathogenic species, such as *Staphylococcus*, *Klebsiella*, *Enterobacter*, *Streptococcus*, and *Candida*, also cause hospital-acquired infections [6].

Several of these pathogenic bacteria and fungi form structures known as biofilms, which are complex amalgamations of cells that are embedded in a matrix of exopolysaccharides [7], providing them with advantages for survival and infection. It has been over 70 years since the first report on biofilms [8], and biofilms remain elusive owing to their complex structure. These biofilms attach to different surfaces, provide advantages for disseminating infections, and are challenging to eradicate. Approximately 90% of microorganisms live in a biofilm, and only 10% are in their planktonic form [9]. If the biofilms dry, then removing them from the surface requires multiple wipes, and the microbial cultures from these biofilms can spread to the next surface [10]. Disinfection and sterilization of surfaces and materials such as silicone elastomers, which are used as silicone fillers/gels, and patches for wound healing are essential, especially in hospitals where the dissemination of infection is higher due to the ease of spreading of infections [11].

Healthcare-associated infections remain a significant challenge due to the persistence of microorganisms on environmental surfaces, particularly in the form of biofilms, which exhibit increased resistance to conventional disinfection methods. The COVID-19 pandemic has further emphasized the importance of effective surface decontamination strategies to limit the transmission of infectious agents in both healthcare and public settings [12]. Ultraviolet (UV) radiation or UV Germicidal Irradiation (UVGI) is used as an effective physical source for sterilization [13]. UV irradiation has been shown to slow biofilm formation [7].

The microorganisms selected in this study represent clinically and environmentally relevant biofilm-forming groups commonly encountered in healthcare and contaminated environments. *B. subtilis* was used as a Gram-positive, spore-forming bacterium, *E. coli* K12 as a representative Gram-negative bacterium, and *C. albicans* as a fungal pathogen. These organisms were chosen as model systems to represent major classes of biofilm-forming microorganisms with differing structural and physiological characteristics, allowing evaluation of UVGI efficacy across diverse microbial types. The aim of this study was not to test the most resistant organisms, but to assess broad-spectrum antimicrobial performance under controlled laboratory conditions [14].

UV radiation is categorized as UV-A (320–400 nm), UV-B (280–320 nm), and UV-C (200–280 nm); UV-C is considered to have germicidal properties [10,15,16,17]. UVGI is a proven physical technique for disinfecting surfaces that are contaminated with microbes. When irradiated with a UV-C source, inactivation is due to photo-biochemical reactions that take place within the cell. The effectiveness of UV-C is attributed to its ability to damage the Deoxyribonucleic Acid (DNA) by altering nucleotide base pairing. Protein molecules, along with DNA and Ribonucleic acid (RNA), absorb UV photons between 200 and 300 nm, with peak absorption at 260 nm, thus destroying the cells [18,19].

The development of a UVGI system is complex, and the challenge lies in the transportation of the system and the manpower involved in the operation of the equipment. Gram-positive and Gram-negative bacteria behave differently and have different infectious and toxigenic effects on humans due to their different cellular mechanism approaches [20]. The UV-C radiation on these bacteria will be different, as the cell walls of Gram-positive and Gram-negative bacteria are different [21].

The motivation for this work stems from several critical research gaps in the current landscape of UVGI technology. Existing commercial UVGI systems are often expensive, rely on proprietary technology, and are typically fixed installations, making them unsuitable for deployment in resource-limited or rural healthcare environments. Additionally, their bulky design and the need for significant manpower to operate and relocate them limit their portability and flexibility, particularly in dynamic settings such as hospitals, clinics, and public spaces. Operational complexity further compounds these challenges, as many systems require trained technicians and involve intricate protocols, which hinder rapid deployment during infectious disease outbreaks. Moreover, while UVGI has demonstrated promising disinfection efficacy, its effectiveness against resilient biofilm-forming bacteria and fungi, especially when tested on clinically relevant substrates such as Petri plates, 96-well microtiter plates, and silicone elastomers across different experimental models, remains inadequately explored [22].

To address these gaps, the present study involves the design and development of a compact, in-house wireless UVGI system integrating hardware and control software for remote operation via a mobile device or computer over a WiFi network. It employs a conventional low-pressure mercury UV-C source with a peak emission at 253.7 nm, which lies close to the DNA absorption maximum and is widely used in germicidal disinfection systems. The antimicrobial efficacy of the system was evaluated using representative biofilm-forming microorganisms, including *B. subtilis* (Gram-positive, spore-forming), *E. coli* K12 (Gram-negative), and a multidrug-resistant clinical isolate of *C. albicans* M-207. The study systematically investigates the effect of UV-C exposure at varying distances and time intervals using CFU enumeration, MTT assay, and SEM analysis. The findings aim to provide a comprehensive assessment of UVGI efficacy against biofilm-associated microorganisms and to evaluate the practical applicability of a wireless UVGI platform for surface disinfection.

In this study, we present the development of a compact, wireless, in-house-designed UVGI system and its evaluation under controlled laboratory conditions. The work is intended as a proof-of-concept investigation that integrates device development with microbial validation, rather than a study of the UVGI method alone. The system was assessed using representative biofilm-forming microorganisms on defined surfaces to evaluate its antimicrobial performance and to validate the response of the test organisms. It is important to note that this study does not aim to demonstrate full-scale environmental or room-level disinfection. Instead, the findings are based on controlled experimental conditions, and factors such as complex surface geometries, shadowing effects, material compatibility, and environmental variability were not addressed. Further studies will be required to evaluate the performance and applicability of the developed system under real-world conditions.

## 2. Results

### 2.1. Device Development

#### 2.1.1. Hardware

**Figure 1:**
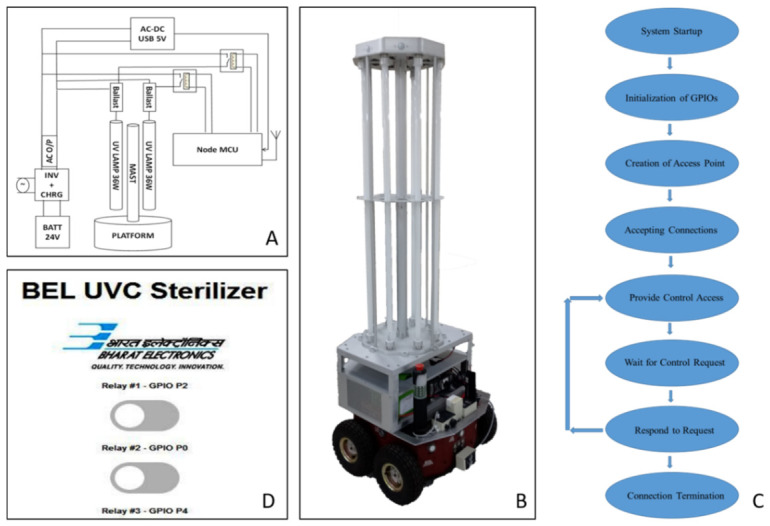
(**A**) A block diagram of the UVGI disinfection system showing the battery, inverter, relay-controlled lamp supply, ESP32/NodeMCU control module, and wireless browser-based interface. (**B**) The mechanical setup of the mobile UVGI platform with eight vertically mounted 36 W Philips UV-C tubes (peak emission at 253.7 nm) supported on an aluminum alloy frame and mounted on a wheeled base. (**C**) A flowchart of system operation and remote control through Wi-Fi. (**D**) Browser-based control page hosted by the ESP32/NodeMCU system for wireless switching of the UV-C lamps.

The UVGI disinfection system consisted of eight UV tubes (Philips, Bengaluru, India), with a peak emission at 253.7 nm, each of 36 W, that were energized with their own ballast. The power supply from the inverter via the AC relay was supplied to each ballast, which is an energizing element that powers the UV tube. The ballast requires a 40 W input to provide 36 W to the UV tube and the remaining for its own power. Hence, a total of 400 W of input power was required, considering the inverter efficacy to be 75–80% and other losses. It was essential to provide AC power as a backup for the system, which was achieved by combining an inverter, charger, and 24 V battery system. The battery was a 50 Ah (ampere-hour) battery from Micronix Ltd. (Mississauga, ON, Canada), a Bengaluru-based micro-, small-, and medium-sized enterprise (MSME). The inverter (Meanwell) was a 24 V DC to 240 V auto-grade AC square-wave converter with an inbuilt short circuit and under/over-voltage protection. The battery was 24 V DC, 50 Ah, and a Li-Ion type battery with a charger. Together, this provides a 3 h power backup. A block diagram of the UVGI system is depicted in Figure 1A, and the overall system specifications are listed in (Table S1).

The eight UV tubes were supported with an aluminum (Alloy 6061-T6) structure (MAST) designed for easy machining without compromising the required strength. The aluminum alloy was further coated with an 18-micron-thick and clear anodic coating to prevent corrosion and reduce damage due to rough handling. The UV tubes were secured in the middle with a centre plate and cylindrical tube holders at their ends. Rubber gaskets with a shore hardness of 65 ± 7 A were fixed between the metal and tube interface to avoid breakage owing to stress. An aluminum extruded hollow rod with a 2-inch outer diameter and a 3 mm was fixed at the centre to provide stiffness to the structure. Four rods with a half-inch diameter were placed around the periphery to improve the stability of the UVGI system. The dosage of UV-C on the surface was measured using a sensor interfaced with a digital interface metre known as a dosimeter. The complete setup of UVGI is shown in Figure 1B.

Irradiance characterization: UV-C irradiance at the sample plane was measured using a factory-calibrated dosimeter (Model RMD Radiometer-814/400C, Opsytec Dr. Gröbel GmbH, Ettlingen, Germany). Measurements were recorded at the experimental exposure distances of 1, 3 and 5 m from the UVGI source, with Petri plates positioned perpendicular to the vertically mounted UV-C tubes. To assess exposure consistency, irradiance was checked at multiple points across the exposure plane, and the variation was found to be within approximately 10%. The measured irradiance values were used for UV dose calculation for each exposure condition.

The UV-C germicidal tubes in the UVGI system with a peak emission at 253.7 nm are standard low-pressure mercury discharge lamps commonly used in germicidal applications because their principal emission lies near 254 nm, close to the wavelength range of maximum nucleic acid absorption. The manufacturer-reported emission profile shows a sharp peak at 253.7 nm with negligible emission below 240 nm; accordingly, the lamps are classified as ozone-free.

#### 2.1.2. Software

The input/output of each relay was connected to Node MCU hardware, which is a SoM based on Espressif System Platform 32 (ESP32), an Advanced RISC Machine (ARM)-based System on Chip (SoC) having inbuilt Wireless Fidelity Media Access Control (WiFi MAC). The MCU SoM node hosts the webpage through which the user can control the system. An AC-DC adapter with a 5 V output was used to power the SoM. The Node MCU SoM had the necessary software to control the system via a web server; therefore, it did not require any secondary software.

When the system was switched on, an access point was created by the hardware that could be connected via WiFi, either on the phone or laptop, to the IP address of the device and copied onto a browser. The browser was then connected to a webpage hosted on the SoM. Using this web browser, the user can control the device wirelessly, without any direct contact with the device. This protects the user from UV radiation and ensures device safety.

The flow of the software is depicted in Figure 1C, which shows the general procedure followed when the system is at the stage of accepting control and providing the connection. Figure 1D shows a snapshot of the web browser.

Thermal and material considerations: In the present study, a dedicated thermal characterization of the UVGI exposure environment was not performed, and therefore the heating effect induced by the system was not quantitatively measured. Likewise, the effect of the UVGI sterilizer on the materials in the surrounding environment was not studied.

### 2.2. Microbial Validation of UVGI Surface Sterilization

#### 2.2.1. Agar Spot Assay

**Figure 2:**
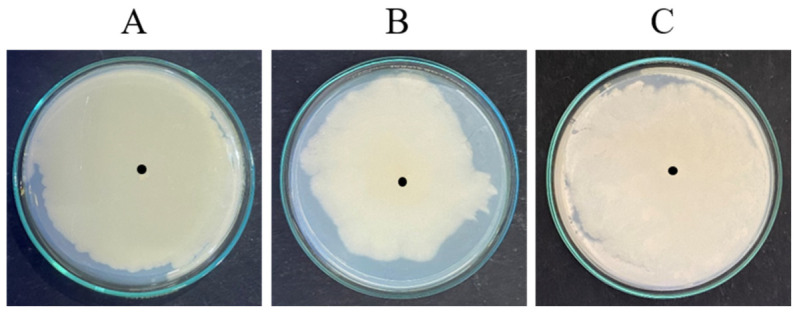
Agar spot assay at 16 h. (**A**) *B. subtilis*, (**B**) *E. coli* K12, and (**C**) *C. albicans* M-207. The black dot is the spot of inoculation. The cultures grew and spread completely, forming a lawn on the Petri plate. This confirmed the ability of the cultures to form a high biofilm.

The biofilm-forming ability of the test organisms *B. subtilis*, *E. coli*, and *C. albicans* M-207 was confirmed by agar spot assay. The agar spot assay was used to assess surface-associated growth and radial expansion from a central inoculation point. This method provides a preliminary indication of the ability of the microorganisms to proliferate and spread across a surface, which is often associated with biofilm-forming potential. After 16 h of incubation, the cultures grew and spread completely, forming a lawn on the Petri plate by point inoculation using an inoculation loop, as depicted in Figure 2. This confirmed the ability of the cultures to form a high biofilm.

#### 2.2.2. Colony-Forming Unit

**Figure 3:**
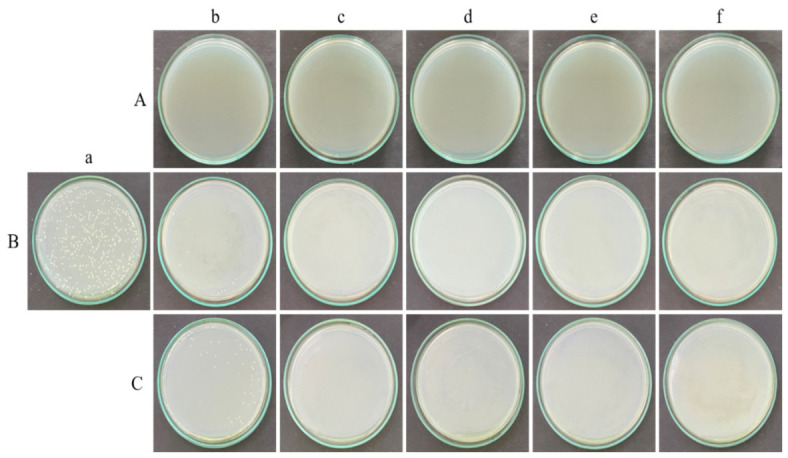
The effect of UV irradiation on *B. subtilis* assessed by CFU assay on nutrient agar plates. The bacterial suspensions were subjected to UV exposure at three distances from the light source: (**A**) 1 m, (**B**) 3 m, and (**C**) 5 m. For each distance, the nutrient agar plates depict (**a**) untreated control cultures and those irradiated for (**b**) 15 min, (**c**) 30 min, (**d**) 45 min, (**e**) 60 min, and (**f**) 90 min. A noticeable decline in colony density with increasing UV dose and closer proximity to the UV source highlights the dose- and distance-dependent bactericidal activity of UV-C treatment against *B. subtilis*.

**Figure 4:**
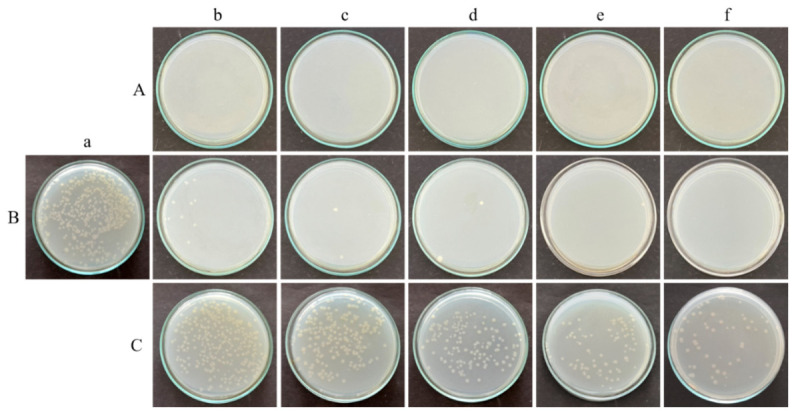
The effect of UV irradiation on ***E. coli*** K12 assessed by CFU assay on nutrient agar plates. The bacterial cultures were exposed to UV light at three different exposure distances: (**A**) 1 m, (**B**) 3 m, and (**C**) 5 m. For each distance, the plates show (**a**) an untreated control (no UV exposure) and samples exposed for (**b**) 15 min, (**c**) 30 min, (**d**) 45 min, (**e**) 60 min, and (**f**) 90 min. A progressive decrease in CFU density with increasing exposure time and proximity to the UV source demonstrates the dose- and distance-dependent bactericidal effect of UV irradiation.

**Figure 5:**
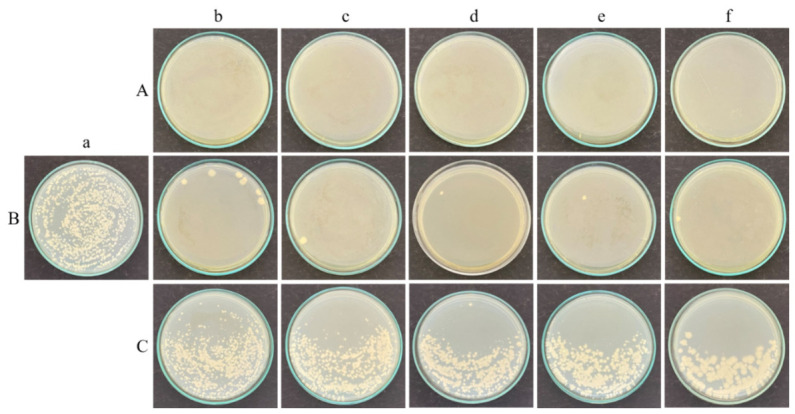
The effect of UV irradiation on *C. albicans* M-207 assessed by CFU assay on nutrient agar plates at (**A**) 1 m, (**B**) 3 m, (**C**) 5 m distances for (**a**) control, (**b**) 15 min, (**c**) 30 min, (**d**) 45 min, (**e**) 60 min, (**f**) 90 min in YEPD media. A decrease in colony count was observed with an increase in exposure time, and also the formation of crescent-shaped aggregates of colonies was observed at all time durations at a distance of 5 m.

The bacterial species *B. subtilis* and *E. coli* K12 were grown in nutrient agar medium and the fungus *C. albicans* M-207 was grown in YEPD medium and irradiated at different time intervals and distances to understand the effect of UV-C radiation emitted by UVGI in inhibiting the growth of these cultures. The CFU analysis reflects the viability of the planktonic cells based on their ability to proliferate and form colonies following UV exposure. UV-C exposure resulted in time- and distance-dependent reductions in viable counts for all the tested organisms, as determined by the CFU analysis, as shown in (Table S2, Figure 3, Figure 4 and Figure 5).

For *B. subtilis*, complete inactivation was observed at 1 m across all exposure durations, with viable counts falling below the limit of detection (≥8.69 log_10_ reduction). At 3 m, a progressive decrease in viability was observed, with reductions increasing from 1.51 log_10_ at 15 min to complete inactivation by 45 min (≥8.69 log_10_ reduction). At 5 m, a gradual reduction in CFU was noted, reaching 2.69 log_10_ reduction at 60 min, and complete inactivation at 90 min.

For *E. coli*, complete inactivation was similarly achieved at 1 m for all time points (≥8.76 log_10_ reduction). At 3 m, a gradual increase in antimicrobial effect was observed, with reductions of 1.71 log_10_ at 15 min, 2.40 log_10_ at 30–45 min, and 2.76 log_10_ at 60 min, followed by complete inactivation at 90 min. At 5 m, reductions were comparatively lower, ranging from 0.38 to 1.02 log_10_ across the tested durations, indicating partial inactivation under these conditions.

For *C. albicans*, complete inactivation was observed at 1 m across all exposure times (≥5.94 log_10_ reduction). At 3 m, substantial reductions were observed, with 2.10 log_10_ reduction at 15 min, increasing to approximately 2.94 log_10_ reduction from 30 min onwards, indicating near-maximal inactivation under these conditions. At 5 m, comparatively modest reductions were observed, ranging from 0.07 to 0.42 log_10_, suggesting limited efficacy at greater distances.

**Figure 6:**
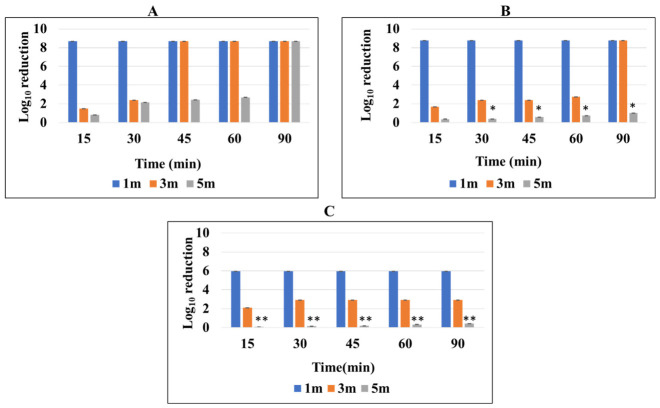
Log_10_ reduction in the CFU counts of (**A**) *B. subtilis*, (**B**) *E. coli*, and (**C**) *C. albicans* following UV-C exposure at different source-to-sample distances and exposure times. Log_10_ reduction was calculated relative to the untreated controls. Values below the LOD are indicated as ≥ log_10_ reduction. The data are presented as mean ± standard deviation (SD). The asterisk represents the significant difference with respect to 1 m distance with * *p* ≤ 0.05, ** *p* ≤ 0.01.

Overall, UV-C exposure demonstrated distance-dependent antimicrobial activity, with maximal efficacy observed at 1 m, and progressively reduced inactivation at 3 m and 5 m. Higher exposure durations resulted in increased microbial inactivation across all organisms, with complete inactivation achieved under higher dose conditions as shown in (Table 1 and Figure 6). The reduction was more pronounced at shorter distances, where higher UV irradiance resulted in higher delivered UV dose for a given exposure time, with statistically significant differences observed compared to 1 m distance (*p* ≤ 0.05–0.01), as determined by two-way ANOVA followed by Tukey’s post hoc test.

#### 2.2.3. Viability of the Cultures Exposed to UVGI

**Figure 7:**
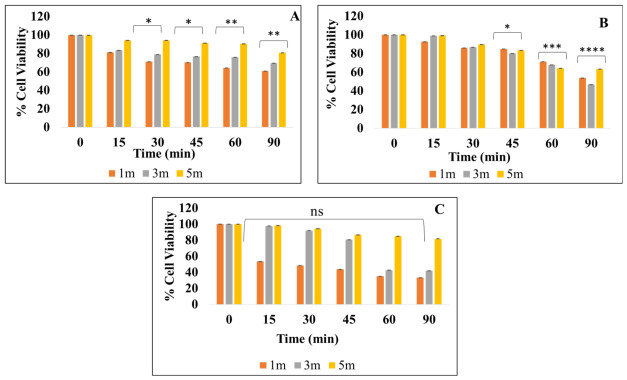
Cell viability analysis using an MTT assay to validate the effect of UV exposure on (**A**) *B. subtilis*, (**B**) *E. coli* K12, (**C**) *C. albicans* M-207 at different distances and time intervals. The asterisk represents the significant difference with respect to the control with * *p* ≤ 0.05, ** *p* ≤ 0.01, *** *p* ≤ 0.001, **** *p* ≤ 0.0001.

The viability of the cultures *B. subtilis, E. coli* K12, and *C. albicans* M-207 on exposure to UV-C was determined using the MTT assay. In *B. subtilis,* the least cell viability of 78.81% was observed at 3 m for 30 min. In *E. coli* K12 at 3 m distances, a low cell viability of 46.97% was observed when exposed to UV for 90 min. *C. albicans* M-207 exhibited 42.71% cell viability at 3 m for 60 min. The cell viability percentage of *B. subtilis*, *E. coli* K12, and *C. albicans* M-207 at different exposure distances and times is shown in (Figure 7, Table 2). The MTT analysis demonstrated a significant decrease in metabolic activity following UV exposure across all the tested conditions. The reduction in cell viability was statistically significant compared to the control group (*p* ≤ 0.05–0.0001), depending on the exposure distance and duration.

In the present study, residual metabolic activity was detected in certain conditions despite a complete loss of culturability. This may be attributed to the presence of metabolically active but non-culturable cells, as well as persister subpopulations that retain enzymatic activity following stress exposure. Additionally, differences in assay sensitivity contribute to this variation, as MTT can detect low levels of cellular metabolism that do not translate into reproductive capacity. Therefore, CFU analysis represents a stricter endpoint for assessing antimicrobial efficacy, while MTT provides complementary information on short-term metabolic activity within the treated microbial population.

#### 2.2.4. Scanning Electron Microscopy (SEM)

**Figure 8:**
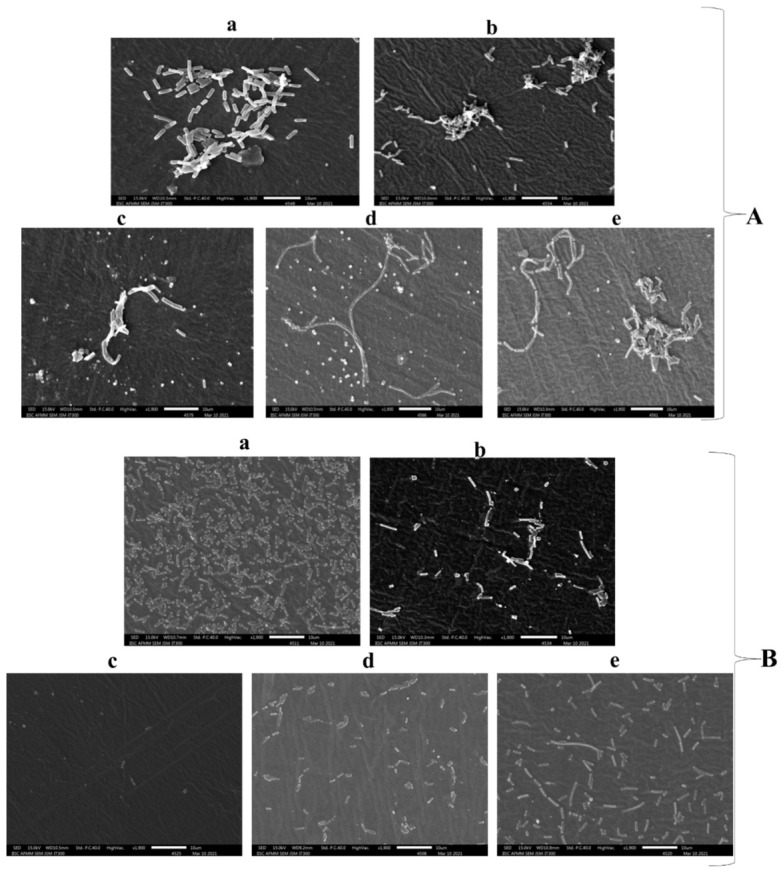

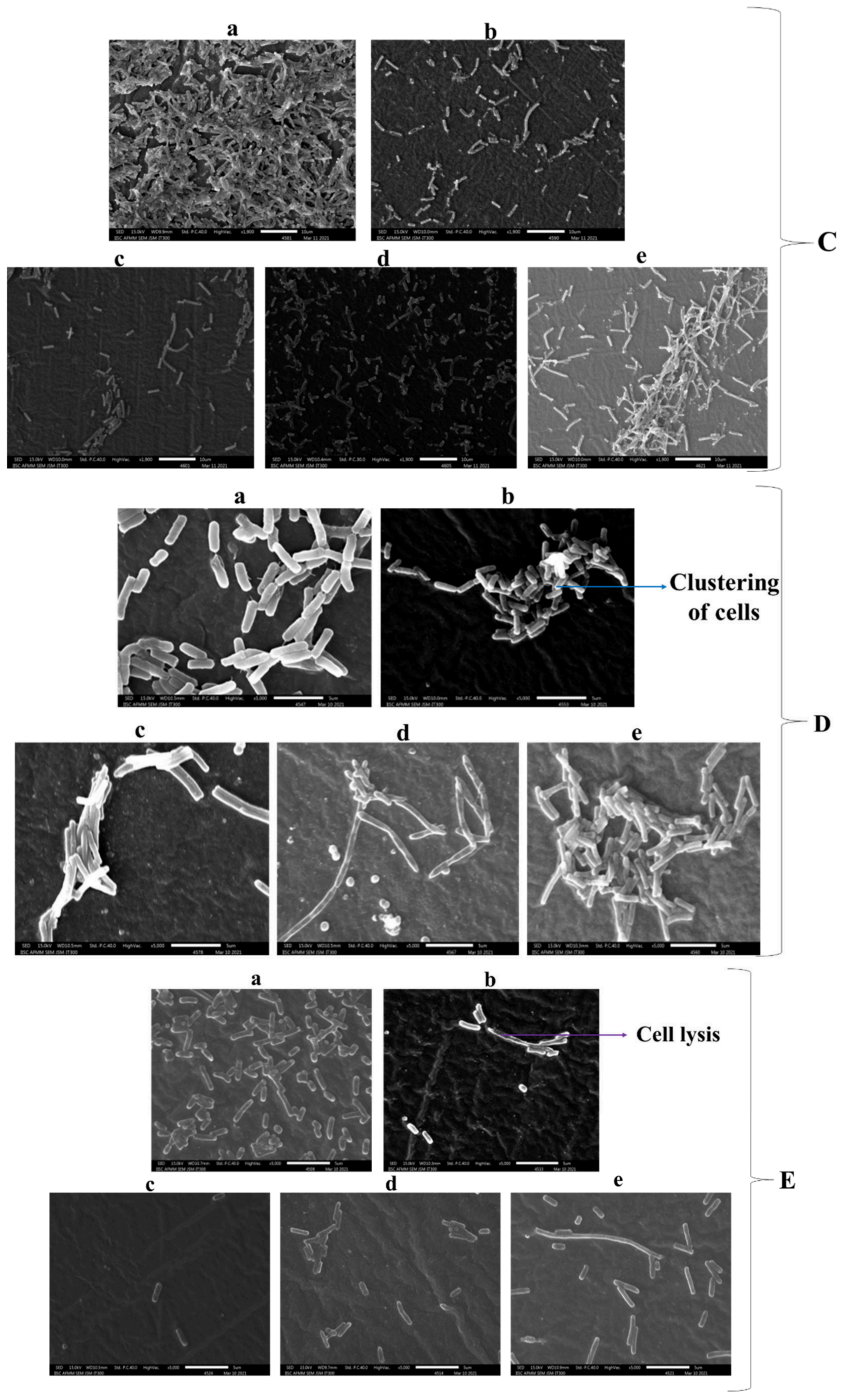

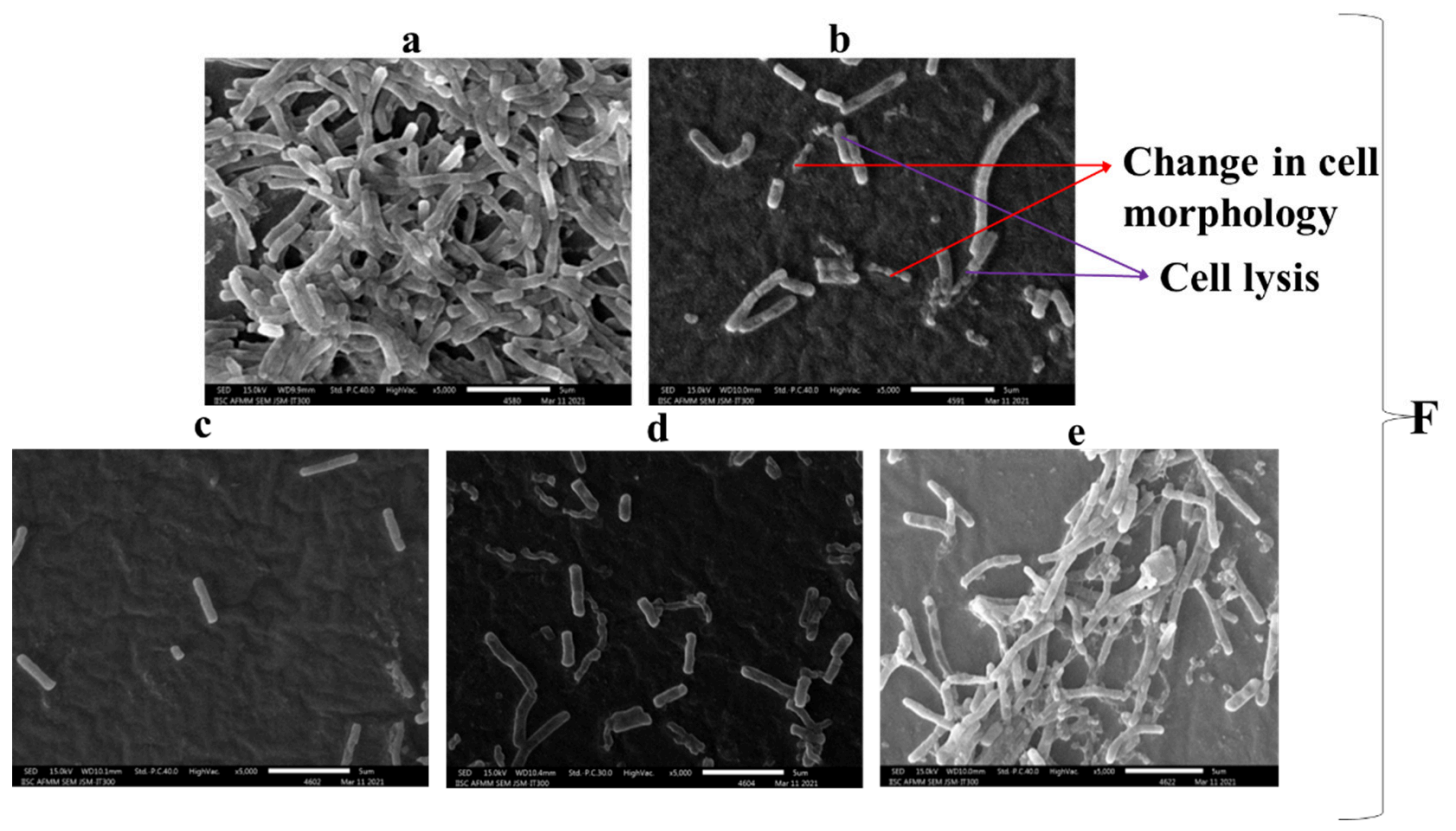
SEM images of representative biofilm-forming microorganisms *B. subtilis* (**A**,**D**), *E. coli* K12 (**B**,**E**), and *C. albicans* M-207 (**C**,**F**) following UV-C exposure. Images at 1900× magnification are shown in panels (**A**–**C**), and corresponding images at 5000× magnification are shown in panels (**D**–**F**). For each organism, representative conditions include: (**a**) untreated control, showing intact biofilm architecture with cells embedded in ECM; (**b**) UV-C exposure at 1 m for 30 min; (**c**) UV-C exposure at 1 m for 60 min; (**d**) UV-C exposure at 3 m for 60 min; and (**e**) UV-C exposure at 5 m for 60 min. UV-C-treated samples exhibit progressive alterations in biofilm structure, including reduction in ECM, decreased cell density, 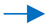 clustering of cells, 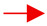 changes in cell morphology and 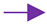 cell lysis, with more pronounced effects observed at shorter distances and longer exposure durations.

Structural characterization using SEM was performed to observe the surface morphology of cultures grown on silicone elastomer discs after UV-C exposure. When the control 1900× and 5000× images of *B. subtilis*, *E. coli* K12, and *C. albicans* M-207 were compared, it was observed that *C. albicans* M-207 formed a very dense biofilm as compared to the other two cultures. Increased aggregation of cells was observed in *B. subtilis* when exposed to UV-C compared to the control. Spore formation in *B. subtilis* was observed at greater UVGI distances. In the case of *E. coli* K12, UVGI was the most effective at a 1 m distance when compared to 3 and 5 m. The dense biofilms of *C. albicans* M-207 were substantially reduced when exposed to the UVGI system for 30 and 60 min at distances of 1 and 3 m, respectively. However, UVGI was not effective in controlling biofilm formation at a distance of 5 m in *C. albicans* M-207. The 1900× and 5000× magnification images of the above-mentioned UVGI exposure studies are shown in (Figure 8).

The SEM images captured at 1900× and 5000× magnifications revealed significant morphological alterations in *B. subtilis*, *E. coli* K12, and *C. albicans* M-207 following UV exposure. The control cells showed intact and typical cellular morphology, whereas the treated cells exhibited surface irregularities, membrane damage, and reduction in the extracellular matrix (ECM) and number of cells, indicating cell death, deformation, and structural collapse. Higher magnification (5000×) highlighted detailed surface disruptions, while lower magnification (1900×) showed reduced cell density and aggregation changes.

## 3. Discussion

Infections are classified as hospital-acquired if they occur within 48–72 h of the visit to the hospital or if they appear within 10 days after hospital discharge (WHO, 2002). Hospital-acquired infections are caused by many microorganisms, predominantly Gram-positive and Gram-negative bacteria, fungi, and viruses [23]. *E. coli* is one of the bacteria that must be tested for household/hospital disinfection. A compact, in-house, easy-to-use, and low-cost UVGI sterilization system was developed in the current study. This system was evaluated for targeted sterilization of hospital wards and operating theatres, which was validated using model bacterial and fungal systems. However, this system can also be used in various other places, such as schools, malls, restaurants, theatres, banks, shopping centres, airports, airplanes, public toilets, and public transport. With little modification to the existing manual portable UVGI system, wall-mounted, table-top, and automated portable models can also be developed [1,2].

Bacteria and fungi form community structures known as biofilms that are difficult to control. These biofilms are composed of microbial cells, proteins, carbohydrates, and enzymes in an ECM. There are channels within the ECM for the transport of nutrients, antimicrobials, waste, etc. There are also void spaces in which nutrients, waste, and antimicrobials are stored. Biofilms formed by these microorganisms protect them by absorbing UV, scavenging ROS, increasing the path length, and scattering UV [9]. Studies have used UV-C radiation on biofilms and have successfully controlled these biofilms [9,24]. However, additional treatments may be required to thoroughly remove the biofilms [10]. If not thoroughly removed, new growth of organisms may occur on the remains of the previous biofilm [25].

Mitigation of microbial contamination and its associated infections by direct exposure to UV irradiation is being practised as a chemical-free approach or in tandem with other sanitation measures to maintain a sterile environment [14,26]. Therefore, UV radiation is a proven technique for chemically free sterilization. However, their effects on biofilm-forming microorganisms are limited. Hypothetically, UV radiation inactivates cells within the biofilm, thus slowing down the process of biofilm formation [9]. The continuous/pulsed mode of UV irradiation has proven to be effective in sterilization [27,28] and reduced biofilm formation [11].

**Figure 9:**
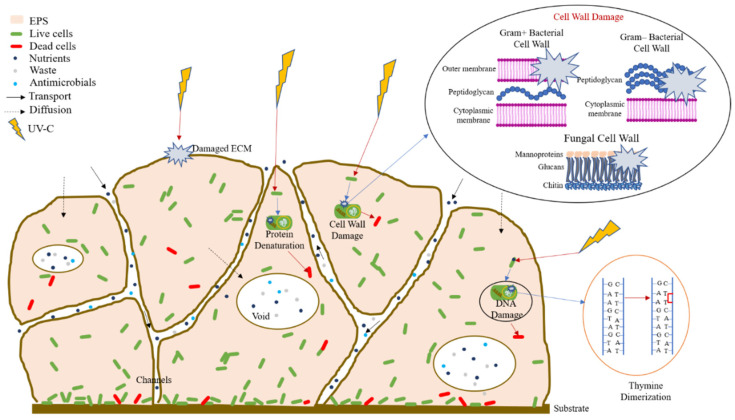
A schematic representation of the proposed UV-C-mediated microbicidal effects on bacteria and fungi, based on the previously reported literature. The figure is illustrative and not derived from the direct experimental evidence in this study.

The UVGI system was effective in controlling biofilm-forming bacteria and yeast. The ECM in the biofilm is damaged by UV-C, causing the disruption and leakage of components. Due to damage to the biofilm ECM, UV-C comes in direct contact with the cell wall, leading to cell damage and death. UV-C photons are also absorbed by intracellular components such as proteins, DNA, and RNA, causing their denaturation/damage. Damage to nucleic acids can occur in the form of double bonds/dimers between adjacent nucleotide [29]. This photochemical damage is known as pyrimidine dimerization, the most common of which is thymine dimerization (Figure 9).

These three microorganisms—*B. subtilis*, *E. coli* K12 and *C. albicans* M-207—were selected because they are all robust biofilm formers and represent different microbial categories commonly encountered in real environments. *B. subtilis* is ubiquitous, can grow in aerobic as well as in anaerobic conditions, is found in soil, water, air, dust, plants, foods, and hospital settings, and can cause secondary nosocomial infections, including wound, respiratory, and systemic infections. *E. coli* K12, an enteric bacterium belonging to the ESKAPEE group (E–*Enterococcus faecium*, S–*Staphylococcus aureus*, K–*Klebsiella pneumoniae*, A–*Acinetobacter baumannii*, P–*Pseudomonas aeruginosa*, E–*Enterobacter* species, E- *Escherichia coli*), represents Gram-negative pathogens commonly involved in environmental contamination and clinical infections. *C. albicans* represents clinically relevant fungal biofilms that are resistant to multiple drugs. Together, these models provide a relevant spectrum of biofilm-forming organisms for evaluating UVGI disinfection, mimicking real-world microbial contamination in hospitals, public spaces, and environmental niches. Three different models were used to study the germicidal action of the UVGI system: a glass Petri plate model to assess viable count (CFU), silicone elastomer discs in a 96-well polystyrene microtiter plate model to assess cell viability (MTT), and silicone elastomer discs in a glass Petri plate model for morphological analysis of the test organisms (using SEM).

Biofilm susceptibility to disinfection is influenced by the stage of maturation. In the present study, 24 h biofilms were used, representing intermediate to mature stages depending on the organism [30]. Therefore, the observed antimicrobial efficacy reflects responses at this defined stage, and variations may occur with more mature biofilms. In this study, sensitivity to UV-C was observed for *B. subtilis*, *E. coli* K12, and *C. albicans* M-207 at different time intervals and distances with cell killing/inhibition observed when the CFUs were counted. A reduction in the number of viable cells with increasing UV dose, determined by irradiance and exposure time, was observed for the bacterial cultures, similar to a published study [28]. However, in the case of *C. albicans* M-207, reduction was observed even at lower UV irradiance when exposure time was increased, resulting in a higher cumulative UV dose. Although there was a definite reduction in colony count, the increase in colony size was striking. Whenever there are fewer colonies, the colony size is large because there is minimum competition for space and nutrients (crowded plates have small colonies). We also observed an interesting phenomenon in *C. albicans* M-207, where at low UV-C, the colonies tended to aggregate in a crescent shape towards one half of the glass Petri plate facing the radiation, appearing partially inhibited. However, this phenomenon has not yet been observed in other cultures. The Petri plate side directly exposed to UV-C formed this crescent-shaped aggregate, whereas the other side had no colonies. This is because of the Petri plate barrier; the UV-C cannot penetrate that side, and the colonies are shielded from the radiation, whereas the colonies on the other side are killed. This also demonstrates that UV-C radiation cannot penetrate glass and polymeric materials, such as polystyrene. The most refractory material for UV-C disinfection is plastic, followed by glass, and stainless steel [31]. A reduction in cell viability was also observed as compared to the control, indicating the sensitivity of the cultures to UVGI, as reported previously [32] in another study.

Microbial inactivation was evaluated using log_10_ reduction in CFU counts, reflecting the extent of reduction in viable cells following UV-C exposure. The degree of inactivation was dependent on UV dose and exposure conditions. Lower exposure conditions resulted in partial reductions (~1–2 log_10_), indicating a decrease in viable cell counts, whereas higher UV doses resulted in greater reductions, with viable counts falling below the LOD under specific conditions, indicating substantial inactivation. The extent of reduction varied among organisms, with *C. albicans* exhibiting comparatively higher susceptibility than the bacterial strains under similar conditions. Overall, UV-C exposure resulted in dose-dependent reductions in microbial viability, with increasing log_10_ reductions corresponding to progressively greater antimicrobial effects.

SEM was performed as a qualitative method to evaluate cell death and morphological changes in the microbes exposed to different UV-C doses at different source-to-sample distances [33,34]. The SEM analysis revealed clear evidence of UV-induced structural damage, including disruption of cell surfaces, reduction in ECM, and decreased cell density across all tested microorganisms. These observations indicate progressive deterioration of biofilm architecture with increasing UV exposure. In the case of *C. albicans* M-207, residual biofilm-like structures were observed under certain exposure conditions, particularly at increased distances from the UV source. This likely reflects incomplete inactivation of the microbial population at lower UV doses. Such observations may be associated with the survival of stress-tolerant or partially damaged cells [35]. Overall, the SEM findings demonstrate that reduced distance and increased exposure time enhance the extent of structural disruption, highlighting the importance of optimizing UV-C dose and exposure conditions for effective microbial inactivation.

A key advantage of the developed UVGI system is its relevance to public health and infection control, particularly in targeting biofilm-forming microorganisms that are often resistant to conventional disinfection methods. The observed reduction in biofilm-associated microbial load under UV-C exposure highlights its potential as a non-chemical disinfection approach. Conducting the study under controlled laboratory conditions enabled precise evaluation of parameters such as UV dose, exposure time, and microbial response, ensuring the reproducibility of the results. In addition, UVGI offers an environmentally favourable alternative by reducing reliance on chemical disinfectants. These findings support further evaluation of UV-based systems for applications in healthcare, water treatment, and food processing environments [36].

The findings of this study align with recent reports demonstrating that UV-C irradiation is a promising strategy for inactivating biofilm-associated microorganisms, although efficacy strongly depends on microbial type, biofilm maturity, and exposure conditions. A study by [36] showed that at 254 nm UV-C irradiation was effective in reducing biofilms of healthcare-associated pathogens on stainless steel surfaces, supporting our observations of biofilm susceptibility under controlled UVGI treatment. Similarly, refs. [37,38] reported that *E. coli* biofilms displayed variable UV sensitivity depending on growth temperature and biofilm maturation. In line with our data on *C. albicans*, ref. [39] demonstrated that portable UV-C devices significantly disrupted *C. albicans* biofilms, highlighting the clinical relevance of targeting fungal biofilms with UV technologies. Furthermore, a recent critical review emphasized that biofilm extracellular matrix composition and heterogeneity often limit complete eradication, as metabolic assays (e.g., MTT) may overestimate viability compared to CFU counts. Collectively, these findings underscore that while UV-C treatment is broadly effective, discrepancies between viability assays reflect complex biofilm physiology and reinforce the value of combining metabolic and culture-based methods, as applied in our study.

Ultraviolet-C (UVC) light, with wavelengths ranging from 200 to 280 nm, has been widely studied for its germicidal properties and is effective against bacteria, fungi, and viruses by causing DNA/RNA damage. Conventional UVC systems, typically operating at 254 nm, are effective for air, surface, and water disinfection; however, direct exposure can be harmful to human skin and eyes, limiting their use in occupied spaces. UVC disinfection systems can be classified as near-UVC (conventional 254 nm) and Far-UVC (207–222 nm), with differences in microbial inactivation efficiency, penetration, and safety profiles. Emerging Far-UVC technology (207–222 nm) has gained attention as a potentially safer alternative. Studies suggest that Far-UVC can inactivate microbes efficiently while penetrating only the outer dead layer of human skin and the tear layer of the eyes, potentially reducing health risks associated with conventional UVC [40,41,42]. While Far-UVC offers a novel approach for continuous disinfection in contaminated public spaces, issues such as lamp cost, dose optimization, and long-term safety must be addressed.

For effective biofilm disruption, especially in applications requiring deep penetration, conventional UVC (254 nm) is currently the more suitable choice. However, Far-UVC (222 nm) holds promise for applications in occupied spaces due to its safety profile, though its efficacy in biofilm disruption is still under investigation [43,44]. Therefore, our study specifically focused on conventional UV-C at 253.7 nm, delivered by low-pressure mercury germicidal lamps, due to its well-established antimicrobial efficacy and broad use in laboratory and environmental disinfection systems, but future work could explore Far-UVC applications as the technology matures.

The current literature reporting UV-C disinfection of biofilms has been systematically compared with our results, and the outcomes are presented in Table 3. Our results, in line with these reports, indicate that biofilms show higher resistance compared to planktonic cells due to the protective EPS matrix. Importantly, the UV dose and exposure time determined whether only surface-associated cells were inactivated or whether deeper biofilm layers were also disrupted. Lower doses mainly reduced surface viability, while higher doses promoted more uniform microbial killing and disruption of the biofilm matrix. These findings emphasize that effective biofilm disinfection requires delivering a sufficient UV dose to not only kill embedded cells but also overcome EPS-mediated protection, which is critical for the design of portable UVGI systems for real-world applications [36].

A limitation of UV disinfection lies in its restricted penetration into complex, multilayered biofilms. The extracellular polymeric substance (EPS) matrix, together with cell density and surface irregularities, attenuates UV radiation and shields embedded cells, allowing some viable microbes to persist despite surface inactivation. This structural protection implies that UV alone may not always ensure complete eradication of mature biofilms, especially in clinical or environmental settings where biofilms can exceed several hundred microns in thickness. Consequently, integrated or sequential approaches may be required. For example, combining UV with chemical disinfectants, photocatalytic agents, or mechanical disruption can enhance matrix breakdown and improve microbial inactivation. Such combined methods may lower the overall energy demand of UV while ensuring more complete biofilm eradication [45]. Our findings underscore the importance of considering these synergistic strategies when developing portable UV disinfection systems for real-world contaminated environments.

Despite the observed efficacy of the UVGI system under controlled laboratory conditions, several limitations should be considered. Laboratory-based results may not fully reflect real-world environments, where factors such as uneven surface geometry, shadowing effects, and the presence of organic matter can reduce UV-C penetration and disinfection efficiency. In addition, naturally occurring biofilms are often more structurally complex and resistant than those developed under laboratory conditions, which may lead to an overestimation of effectiveness [46].

Furthermore, the use of UV-C radiation requires appropriate safety precautions due to potential health risks associated with direct exposure. The effectiveness of UVGI systems also depends on proper calibration, positioning, and consistent operational practices. In practical applications, considerations such as installation cost, maintenance, and system optimization may influence large-scale implementation. Therefore, further validation under real-world conditions is necessary to fully establish the applicability of the developed system [47].

During the exposure conditions investigated in this study (1–5 m, 15–90 min), no visible deformation, melting, discoloration, or structural damage was observed in the laboratory materials used, including Petri plates, polystyrene microtiter plates, silicone elastomer discs, and the aluminum support components. The antimicrobial activity observed in this work is attributed primarily to the germicidal UV-C irradiation from the low-pressure mercury lamps operating at 253.7 nm rather than to a thermal mechanism. However, because repeated UV-C exposure can affect certain surrounding materials, especially UV-sensitive polymers, elastomers, coatings, and painted surfaces, material compatibility assessment is recommended before routine deployment in real-world environments [36].

In addition to safety considerations, the potential impact of UV-C exposure on surrounding materials should be considered. Prolonged or repeated exposure to UV-C radiation is known to cause degradation of certain materials, particularly polymers, leading to surface discoloration, brittleness, or reduced mechanical integrity. In the present study, no visible damage to laboratory substrates was observed under the applied exposure conditions. However, material compatibility was not systematically evaluated, and the effects may vary depending on exposure duration, UV dose, and the nature of the material. Therefore, further studies are required to assess the long-term impact of UV-C exposure on commonly used environmental surfaces and materials [48,49].

The use of polystyrene microtiter plates and silicone elastomer discs as model systems to study the UVC germicidal effect on biofilm-forming bacteria and fungi enhances the clinical relevance of our findings. Polystyrene is widely used in healthcare settings such as diagnostic and laboratory devices (Petri dishes, microtiter plates), in vitro diagnostic components (elisa plates, diagnostic assay cartridges), disposable medical consumables (syringe barrels, blood and urine collection tubes), packaging and sterile barriers (blister packs for medical devices, sterile trays for surgical instruments ), cell culture and tissue engineering, and research-to-clinical translation (tissue culture-treated polystyrene plates and dishes, cell culture inserts) [50,51,52]. Silicone elastomer is commonly employed in medical devices such as indwelling catheters (urinary catheters, central venous catheters), respiratory devices (endotracheal tubes, tracheostomy tubes), implantable devices (silicone breast implants, pacemaker lead insulation), neurosurgical devices (ventriculoperitoneal shunts, external ventricular drains), wound and drainage systems (surgical drains, silicone wound dressings), ophthalmic devices (intraocular lenses, silicone punctal plugs), prosthetics and soft implants (facial prostheses, joint spacers) [53,54,55]. Since these materials are prone to biofilm-associated contamination in healthcare settings, demonstrating effective UV disinfection on these substrates provides valuable translational insight into potential real-world applications. However, it is important to recognize that real-world environments present additional challenges such as surface irregularities, complex geometries, shadowed zones, and heterogeneous organic loads, which can all limit UV penetration and efficacy. When UVC penetration is limited, combined strategies such as mechanical cleaning, chemical disinfectants, or photocatalysis may be required to ensure complete removal of resilient biofilms [56].

Petri plates, 96-well microtiter plates, and silicone elastomer were used as experimental models to evaluate shielding/barrier and shadowing effects during UVGI exposure. At 1 m and 15 min (corresponding to doses of 600.3, 576, and 697.5 mJ/cm^2^), complete inactivation was observed, corresponding to ≥8.69, ≥8.76, and ≥5.94 log_10_ reductions for *B. subtilis*, *E. coli*, and *C. albicans*, respectively (below the LOD). The limited penetration of UV-C through glass and most plastics such as polystyrene has important implications for the design and effectiveness of UVGI systems. Since germicidal UV-C is largely absorbed by these materials, microorganisms shielded behind barriers or embedded in multilayer biofilms may not be effectively inactivated. Recent studies highlight this challenge and potential solutions; for example, thin polystyrene surfaces allow limited UVC transmission while maintaining structural integrity and chamber designs incorporating reflective surfaces [57] and UV-transparent materials can significantly improve dose uniformity and microbial inactivation [58,59]. Modern reviews also emphasize the importance of accounting for optical losses and shielding effects when designing UV-LED and UV-C lamp systems for practical applications [60]. These findings underscore that careful selection of materials, exposure geometry, and reflective surfaces is essential to translate laboratory results into reliable, real-world UVGI applications in healthcare, water treatment, and environmental sanitation.

Infectious disease outbreaks have led to an understanding of the importance of cleanliness, disinfection, and sterilization. Microbes are important and fundamental forms of life from the early stages of Earth’s evolution and therefore can survive under any environmental conditions and acclimatize due to adaptive mutations [61]. Therefore, this study was conducted to determine the effectiveness of UVGI as a microbicidal platform. The UVGI disinfectant system was developed indigenously and is easy to operate and transport, and economical. It has been shown to effectively sterilize bacterial and fungal cultures, with a direct correlation between the exposure distance and time. Therefore, this system can be used for effective disinfection or sterilization of medical devices and hospital areas, such as waiting rooms, laboratories, operation theatres, isolation rooms, emergency rooms, schools, malls, restaurants, homes, and the food industry. The developed UVGI system can be scaled up, modified, and programmed according to requirements to ensure minimum infection risk.

## 4. Materials and Methods

### 4.1. Development of the UVGI Surface Sterilizer System

Commercially available UVGI equipment has the disadvantage of having a complex structure, being fixed and suitable only for targeted and specific room sterilization, and having proprietary technology; therefore, servicing is also expensive, especially affecting rural health centres around the world. The UVGI system developed in this study is a simple assembly setup that is convenient, portable, and can be operated in wireless mode. The UVGI system was developed with two basic components: hardware and software. The hardware component consists of UV tubes, chargers, batteries, inverters, Node Microcontroller Unit (MCU) hardware, an aluminum structure to hold everything in place, and rubber gaskets.

UV dose values are calculated as: UV dose (mJ/cm^2^) = UV irradiance (mW/cm^2^) × exposure time (s).

Example: *E. coli* UV dose values are calculated as follows:

- At 1 m, 0.64 mW/cm^2^, 15 min → 576 mJ/cm^2^;
- At 3 m, 0.177 mW/cm^2^, 30 min → 318.6 mJ/cm^2^;
- At 5 m, 0.032 mW/cm^2^, 90 min → 172.8 mJ/cm^2^.

The dosimeter is factory calibrated.

A dosimeter (model: RMD Radiometer-814400C; make: Opsytec, Dr. Gröbel Gmbh, Ettlingen, Germany) was used as an external system to measure the UV irradiance. The software is an in-built application in the Node MCU System on Module (SoM).

- Lamp type and peak wavelength: Philips UV-C germicidal tubes, 36 W, with a peak emission at 253.7 nm.
- Irradiance uniformity: The irradiance was measured at sample positions (1 m, 3 m, 5 m) using a calibrated dosimeter, and uniformity across the exposure plane was verified by measuring at multiple points, showing <10% variation in irradiance.
- Lamp lifetime: The manufacturer specifies an operational lifetime of ~9000 h at 80% rated output.
- Emission characteristics: The tubes have a sharp emission peak at 253.7 nm, with negligible emission below 240 nm, making them ozone-free.
- The UVGI system was developed considering the requirements of reduced manpower, portability, and simple applications. The UVGI was structurally assembled using locally sourced materials that can be easily mass-produced if needed. Any person with a basic knowledge of electronics can assemble it; hence, the labour hours and cost for assembling is low. This device can be used for sterilization and basic training.

In this study, lamp power refers to the electrical rating of the UV-C source, UV irradiance refers to the radiant power incident on the sample surface per unit area (mW/cm^2^), and UV dose refers to the time-integrated irradiance delivered to the sample (mJ/cm^2^). UV dose was calculated from measured irradiance and exposure time for each exposure condition. Exposure time, irradiance, and dose were therefore treated as distinct parameters throughout the study.

#### Device Operation

The UVGI system was operated using a wireless control interface that enabled remote switching of UV-C lamps through a relay-based mechanism. The experiments were conducted under controlled laboratory conditions with predefined exposure durations and distances. All procedures were performed following established UV-C safety protocols. The irradiation area was cleared of personnel prior to operation, and access was restricted during exposure. The operators used appropriate personal protective equipment (PPE), including UV-blocking goggles, gloves, and protective clothing. Measures were also taken to minimize direct exposure and ensure safe handling of the system. Detailed operational steps and safety specifications are provided in the Supplementary Information.

Ultraviolet-C (UV-C) irradiation at 253.7 nm is widely recognized as a non-thermal (“cold”) disinfection method, where microbial inactivation occurs primarily through photochemical damage to nucleic acids rather than heat. However, we acknowledge that a small fraction of UV energy may be absorbed and converted into heat, leading to a slight increase in temperature during system operation. Under the operating conditions used in this study (low-pressure mercury lamps, exposure distances of 1–5 m, and exposure durations in the range of minutes), any temperature increase is expected to be minimal (typically within ~0.5–3 °C and unlikely to exceed ~5 °C). Such changes are insufficient to contribute significantly to microbial inactivation.

We also note that the extent of heating can vary depending on material properties, including UV absorption characteristics, surface texture, and thermal conductivity, with more absorptive or insulating materials potentially exhibiting slightly higher temperature increases. Although direct temperature measurements were not performed in the present study, all experiments were conducted under ambient laboratory conditions (~22–25 °C), and no visible or noticeable thermal effects were observed during operation. Therefore, the antimicrobial effects reported are attributed predominantly to UV-C irradiation rather than thermal contributions.

### 4.2. Microbial Strains

Three different biofilm-forming model microbial systems were selected for this study. A Gram-positive spore-forming bacteria, *Bacillus subtilis* MTCC 441 (ATCC-6633), and a Gram-negative non-spore-forming bacterium, *Escherichia coli* K12 (MTCC–1302), which is motile with peritrichous flagella, was procured from IMTECH, Chandigarh, India. The Yeast: *C. albicans* M-207, a fluconazole and caspofungin multidrug-resistant clinical isolate from the umbilical vein catheter of a female baby with invasive *Candidiasis*, was provided by the Department of Microbiology, M S Ramaiah Medical College and Teaching Hospital, Bengaluru, India. No human subjects were directly involved in the study; hence, ethical clearance/consent was not required.

We previously used *C. albicans* M-207 clinical isolate as a model system in earlier studies. The growth conditions of *Candida* species, including *C. albicans* M-207, have been optimized using design of experiments (Response Surface Methodology (RSM)) [62]. The growth media for *C. albicans* M-207 and *E. coli* ATCC 39936 in a polymicrobial association have also been optimized using RSM [63]. *C. albicans* M-207 was found to be multidrug-resistant (MDR) (Fluconazole and Caspofungin); hence, studies on the control of *C. albicans* M-207 [64] and its polymicrobial interaction with *E. coli* [65] have been conducted with aqueous spice extracts (Garlic, Indian gooseberry, and clove).

### 4.3. Growth Conditions

*B. subtilis* and *E. coli* K12 were streaked on nutrient agar and incubated at 37 °C for 24 h, whereas Yeast Extract Peptone Dextrose (YEPD) agar was used to maintain *C. albicans* M-207 at 32 °C for 24 h. Colonies were picked from these plates and used for further studies. The culture plates were subcultured every 15 days. Glycerol stocks (15% *v*/*v*) of the cultures were maintained at −20 °C for short-term storage, and the mother cultures were maintained at −86 °C.

The microbial inoculum was standardized to approximately 1 × 10^6^ CFU/mL using hemocytometer-based cell counting. The cell suspensions were enumerated using a hemocytometer, and the concentration was adjusted accordingly by dilution with sterile medium to achieve the desired cell density. This standardized inoculum was used to ensure reproducibility across experiments [66].

### 4.4. Substrate Material

In two of the test models, silicone elastomer discs were used as substrates to grow the bacteria and yeast cultures, as this material is widely used in hospitals in medical devices such as catheters, liners for prostheses, valves, tubing, and long-term implants owing to its biocompatibility. Studying the effect of UV-C on cells grown on silicone elastomer materials will be helpful in controlling the formation of biofilms on medical devices made of this material [67].

### 4.5. Agar Spot Assay

The agar spot assay was performed to qualitatively assess microbial growth and spreading behaviour. Actively growing cultures of *B. subtilis*, *E. coli* K12, and *C. albicans* M-207 were used as inoculum. A loopful of each culture was aseptically transferred and inoculated at the centre of sterile tryptic soy agar (TSA) plates. The plates were incubated at 37 °C in a thermostatic incubator for 16 h, corresponding to the stationary phase for the bacterial cultures and the late exponential to early stationary phase for *C. albicans*. After incubation, the extent of colony spread from the point of inoculation was qualitatively assessed. All the experiments were performed in triplicates [68,69].

For the agar spot assays, the microbial cultures were grown for 16 h prior to inoculation, corresponding to the stationary phase for the bacterial strains (*B. subtilis* and *E. coli* K12) and the late exponential to early stationary phase for *C. albicans*. For the biofilm-based assays (MTT and SEM), the biofilms were grown on a 96-well microtiter plate and silicone elastomer discs for 24 h under static conditions prior to UV-C exposure. At this time point, the *E. coli* biofilms represented an intermediate to early mature stage, the *B. subtilis* biofilms were approaching maturity, and *C. albicans* formed mature biofilms [64].

### 4.6. Preparation of Inoculum

The pre-inoculum was prepared from subcultured plates showing biofilm growth and a loopful of the culture was inoculated in the respective broth media (nutrient broth for *B. subtilis* and *E. coli* K12 and YEPD for *C. albicans* M-207) in a test tube and incubated for 16 h in an incubator shaker at 100 rpm at 37 °C.

Though the pre-inoculum was prepared in liquid media, we maintained the shaker speed of 100 rpm to control and promote biofilm growth. Biofilm formation has been previously optimized. While shear forces in liquid culture limit surface attachment, microcolonies and suspended aggregates (non-attached biofilm fragments) are still present. In static or low-shear liquid cultures, biofilms form on the bottom and sides of wells or tubes, rather than freely suspended in the medium. Thus, the pre-inoculum retains biofilm characteristics and, when added onto silicone elastomer discs, it develops into mature surface biofilms. Therefore, the UV-C disinfection experiments were performed on established biofilms, consistent with the study objectives and relevant to real-world biofilm-associated microbial contamination [66].

### 4.7. Glass Petri Plate Model to Assess Viable Count of UVGI Irradiated Cultures—CFU

The microbial inoculum for the UVGI experiments was standardized to 1 × 10^6^ CFU/mL for all organisms to ensure consistent initial cell loading and reproducible formation of surface-attached communities under experimental conditions. Serial dilutions of actively growing cells were prepared using a slightly modified protocol [70]. A 100 µL volume of 10^−5^ dilution of the bacterial cultures and 10^−2^ dilution of the yeast culture were added to the NA and YEPD glass Petri plates, respectively, and spread evenly on the agar surface using a sterile spreader. A lower dilution was employed for the yeast culture to obtain countable colonies within the acceptable range (30–300 CFU per plate). This adjustment accounts for the differences in cell size, aggregation behaviour, and growth characteristics of the yeast compared to the bacterial cultures, which can otherwise result in lower apparent CFU counts at higher dilutions [71].

The Petri plates were placed inside the laminar airflow (LAF) chamber and exposed to UV-C irradiation from the UVGI system at source-to-sample distances of 1, 3, and 5 m with a UV dose of 600.3, 576 and 697.5 mJ/cm^2^. The UV exposure experiments were conducted under laminar air flow conditions at ambient room temperature (approximately 22–25 °C) to maintain aseptic conditions. The Petri plates were placed perpendicular to the tubelight mounted vertically on the robot and parallel to the LAF bench. The UV irradiance was measured at all distances using a calibrated UV dosimeter. The plates were exposed to UV-C for 15, 30, 45, 60, or 90 min at each distance. The control sets were maintained in parallel. The control sets were plates with microbial culture without UV exposure. The plates were inoculated with the culture and incubated in the incubator, just like the ones after the UV exposure [72]. All the experiments were performed in triplicates. After each exposure, the plates were sealed and incubated at 37 °C for 18 h. After incubation, the colonies were counted and Log_10_ (CFU/mL) values were calculated using the formula as shown in (Equation (1)), and corresponding log_10_ reductions were determined using (Equation (2)). The LOD of the CFU assay was calculated as shown in (Equation (3)). Samples with no detectable colonies were considered below the LOD, and corresponding log_10_ reductions were expressed as ≥values based on the detection limit.

log_10_ (CFU/mL) = Number of colonies × dilution factor/volume of culture plated (1)

Log_10_ reduction in microbial count following UV-C exposure was calculated using:

Log_10_ reduction = log_10_(A) − log_10_(B) (2)

where *A* represents the initial viable count (control, before UV exposure) and *B* represents the viable count after UV exposure.

The LOD of the CFU assay was calculated as:

LOD (CFU/mL) = Minimum detectable colonies/Plated volume (mL) × Dilution factor(3)

### 4.8. Silicone Elastomer Disc in 96-Well Polystyrene Microtiter Plate Model to Assess Viability of the Cultures Exposed to UVGI—MTT Assay

Sterile 5 mm diameter silicone elastomer discs were placed in separate wells of 96-well flat-bottom polystyrene microtiter plates. The inoculum (100 µL) was added to each well of the microtiter plate and incubated at 37 °C for 90 min. After 90 min, 1 × phosphate-buffered saline (PBS) was added, and the plates were exposed to UV-C for 15, 30, 45, 60, and 90 min at 1, 3, and 5 m distances. Control plates of microbial culture without UV-C irradiation were maintained in parallel. During UV exposure, the samples were maintained in PBS, which provides an optically clear, isotonic medium with minimal UV absorption, thereby ensuring delivery of a defined UV dose while minimizing attenuation by organic material. After exposure, the PBS was gently removed to remove non-adherent planktonic cells, and 100 µL of fresh growth medium was added to recover and resuspend the remaining attached biofilm cells. The plates were then incubated for 24 h at 37 °C and then, the MTT assay was performed [73]. After the incubation period, the medium was discarded and the wells were washed with PBS. A 50 µL volume of 5 mgmL^−1^ MTT solution was added to each well and incubated for 3 h in the dark. After incubation, 100 µL of acidified isopropanol was added to each well to dissolve formazan crystals and incubated for 10–20 min with gentle shaking. The wells were mixed thoroughly, 100 µL of the mixture was transferred to a fresh well, and the absorbance was read at 540 nm using a Synergy HT microtiter plate reader [74].

### 4.9. Silicone Elastomer Discs in Glass Petri Plate Model for Morphological Analysis of the Test Organisms on Irradiation—SEM

The biofilms were established on sterile silicone elastomer discs using standardized inoculum. The cultures were revived from stock, subcultured on NA or YPD plates, and loopful growth was transferred into 5 mL NB or YPD broth (pre-inoculum) and incubated overnight (37 °C for *E. coli* and *B. subtilis* and 32 °C for *C. albicans*). The pre-inoculum was diluted 1:60, adjusted to 1 × 10^6^ cells/mL, and added onto the discs under identical incubation conditions. After growth, non-adherent cells were gently removed by PBS washing, leaving only surface-attached biofilms. This procedure ensured reproducible inoculum density and minimized variability in baseline biofilm formation across replicates [75].

Serially diluted cultures of bacteria and yeast were prepared in nutrient broth and YEPD broth, respectively, and poured into Petri plates containing 15 mm diameter silicone elastomer discs. The Petri plates with silicone elastomer discs were exposed to UV-C for different durations and distances. The Petri plates were then incubated overnight. The control set was maintained in parallel. After incubation, the discs were washed with PBS and fixed using 4% glutaraldehyde for 1 h. Subsequently, the discs were washed with PBS before sequential dehydration in an ethanol series: 70% for 10 min, 95% for 10 min, and 100% for 20 min [64,76]. The discs were then air-dried completely, coated with gold under vacuum, and visualized at 1900× and 5000×, 15 kV using a JEOL IT 300 Scanning Electron Microscope at AFMM, Indian Institute of Science, Bengaluru.

### 4.10. Statistical Analysis

Three independent tests were performed to ensure the reliability and reproducibility of the data. All the experimental data are expressed as the mean ± standard deviation. Sextuplicates were maintained for each of the experiments involving microtiter plates, and triplicates were maintained for the other experiments. For log_10_ reduction, the significance of 3 m and 5 m with respect to 1 m was determined. For cell viability, the data are presented as a percentage of the control, and the significance with respect to the control is presented. Two-way analysis of variance (ANOVA) and Tukey’s multiple comparison tests were performed using GraphPad Prism 9.

Linear regression analysis and the calculation of the coefficient of determination (R^2^) are commonly used statistical tools to evaluate the strength and linearity of a relationship between two variables. However, in microbiological experiments, especially those involving microbial growth, biofilm formation, disinfection kinetics, biological variability, non-linear growth dynamics, and environmental influences often make it difficult to obtain a high R^2^ value (close to 1). Microbial responses are inherently complex and influenced by multiple factors that do not always follow a simple linear trend, so linear regression is not typically suitable for interpreting experimental outcomes in such systems.

## 5. Conclusions

In this study, a compact, wireless, in-house-developed UV-C-based UVGI system was evaluated for its antimicrobial efficacy against representative biofilm-forming bacterial and fungal pathogens under controlled laboratory conditions. The system demonstrated dose-dependent microbial inactivation, with substantial reductions in viable cell counts and clear disruption of biofilm architecture, as evidenced by the CFU, MTT, and SEM analyses. The findings indicate that UV-C exposure can effectively reduce microbial viability across both planktonic and biofilm-associated models, with variations observed depending on organism type and exposure conditions. However, this study was conducted under controlled laboratory settings using defined biofilm stages, and factors such as complex surface structures, environmental variability, and material interactions were not addressed. Therefore, while the results demonstrate the proof-of-concept efficacy of the developed UVGI system, further studies are required to evaluate its performance under real-world conditions and to assess long-term operational safety and material compatibility. Such investigations will be essential for validating its applicability in clinical and environmental disinfection settings.

## Figures and Tables

**Table 1:** Log_10_ CFU/mL and corresponding log_10_ reduction in *B. subtilis, E. coli*, and *C. albicans* following UV-C exposure at different source-to-sample distances and time intervals. Values below the limit of detection (LOD) are indicated as <LOD, and corresponding reductions are expressed as ≥values.

		1 m		3 m		5 m	
Culture	Time (min)	(log_10_ CFU/mL)	(log_10_ Reduction)	(log_10_ CFU/mL)	(log_10_ Reduction)	(log_10_ CFU/mL)	(log_10_ Reduction)
***B. subtilis***	0	8.694 ± 0.007	0	8.694 ± 0.007	0	8.694 ± 0.007	0
	15	<LOD	≥8.694	7.188 ± 0.060	1.51	7.875 ± 0.025	0.82
	30	<LOD	≥8.694	6.301 ± 0.000	2.39	6.540 ± 0.088	2.15
	45	<LOD	≥8.694	<LOD	≥8.694	6.239 ± 0.337	2.45
	60	<LOD	≥8.694	<LOD	≥8.694	6.000 ± 0.000	2.69
	90	<LOD	≥8.694	<LOD	≥8.694	<LOD	≥8.694
***E. coli***	0	8.756 ± 0.01	0	8.756 ± 0.01	0	8.756 ± 0.01	0
	15	<LOD	≥8.756	7.047 ± 0.10	1.71	8.378 ± 0.03	0.378
	30	<LOD	≥8.756	6.360 ± 0.10	2.396	8.342 ± 0.04	0.414
	45	<LOD	≥8.756	6.360 ± 0.10	2.396	8.157 ± 0.08	0.599
	60	<LOD	≥8.756	6.00 ± 0.00	2.756	7.995 ± 0.02	0.761
	90	<LOD	≥8.756	<LOD	≥8.756	7.742 ± 0.09	1.014
***C. albicans***	0	5.941 ± 0.005	0	5.941 ± 0.005	0	5.941 ± 0.005	0
	15	<LOD	≥5.941	3.845 ± 0.000	2.096	5.867 ± 0.003	0.074
	30	<LOD	≥5.941	3.000 ± 0.000	2.941	5.787 ± 0.008	0.154
	45	<LOD	≥5.941	3.000 ± 0.000	2.941	5.731 ± 0.002	0.21
	60	<LOD	≥5.941	3.000 ± 0.000	2.941	5.599 ± 0.004	0.342
	90	<LOD	≥5.941	3.000 ± 0.000	2.941	5.519 ± 0.059	0.422

**Table 2:** Cell viability of *B. subtilis*, *E. coli* K12 and *C. albicans* M-207 at different source-to-sample distances and exposure times.

Culture	Time	Distance and Mean Cell Viability (%) ± Standard Deviation		
		1 m	3 m	5 m
***B. subtilis***	0	100.000 ± 0.006	100.000 ± 0.006	100.000 ± 0.006
	15	81.041 ± 0.006	83.271 ± 0.005	94.424 ± 0.006
	30	71.004 ± 0.005	78.810 ± 0.008	94.424 ± 0.016
	45	70.260 ± 0.014	76.580 ± 0.012	91.450 ± 0.007
	60	64.312 ± 0.013	75.836 ± 0.012	90.706 ± 0.010
	90	60.967 ± 0.006	69.517 ± 0.003	80.669 ± 0.016
***E. coli*** **K12**	0	100.000 ± 0.015	100.000 ± 0.015	100.000 ± 0.015
	15	92.647 ± 0.007	98.875 ± 0.033	99.394 ± 0.007
	30	86.159 ± 0.001	86.938 ± 0.016	89.792 ± 0.038
	45	85.121 ± 0.030	80.190 ± 0.004	83.564 ± 0.040
	60	71.367 ± 0.015	67.993 ± 0.029	64.100 ± 0.006
	90	53.979 ± 0.029	46.972 ± 0.009	63.581 ± 0.020
***C. albicans*** **M-207**	0	100.000 ± 0.114	100.000 ± 0.114	100.000 ± 0.114
	15	53.559 ± 0.061	97.825 ± 0.105	98.392 ± 0.052
	30	48.522 ± 0.009	92.220 ± 0.064	94.703 ± 0.088
	45	43.698 ± 0.078	80.941 ± 0.038	86.900 ± 0.114
	60	35.257 ± 0.038	42.705 ± 0.058	85.268 ± 0.052
	90	33.412 ± 0.033	42.067 ± 0.0490	81.863 ± 0.055

**Table 3:** Comparative studies on UVGI efficacy against biofilm-forming microorganisms with the existing literature.

Organism(s)	Inoculum Density and Material	UV Source Type and Mobility	UV Dose (mJ/cm^2^), Distance and Time of Exposure	CFU (Long-Term Viability Assessment)	MTT (Metabolic Activity/Short-Term Viability Assessment)	References
*B. subtilis* *E. coli* K12 *C.albicans* M-207	1 × 10^6^ cells/mL; biofilm formed on Petri plate, 96 well microtiter plate and silicone elastomer discs	UV tubes (Philips-36 W) UV-C (253.7 nm) wireless, portable, laptop/mobile-connectable	576 (1 m), 318.6 (3 m), 172.8 (5 m) 1–5 m, 15–90 min	1 m/15 min → Complete inactivation was observed (≥log_10_ reduction; below LOD) *B. subtilis* → ≥8.694 log_10_ reduction *E. coli* → ≥8.756 log_10_ reduction *C. albicans* → ≥5.941 log_10_ reduction	*B. subtilis* −64% viability (1 m, 60–90 min); *E. coli* 47% (1 m, 90 min); *C. albicans* < 50% (1 m all intervals, 3 m at 60–90 min)	Present study
*S. aureus*, *E. coli*, *P.aeruginosa*, *C.albicans*	Biofilm formed on stainless steel discs	UV-C (254 nm) Low-pressure mercury lamp; wired; bench-top unit	467.8–946.7	>3 log_10_ reduction in bacterial biofilms; *C. albicans*—3.17 log reduction	Not reported	[36]
*E. coli* biofilms (different maturation: 1d @ 37 °C, 3d @ 25 °C, 5d @ 15 °C)	Biofilms matured at different temps, ~10^6^ CFU/cm^2^	UV-C (254 nm), Low-pressure mercury lamp; wired; lab chamber	~30–180 s (lamp output dependent)	Young biofilms → 3.5 log reduction; Mature biofilms → 1.8 log reduction	Not reported	[37]
*Clostridioides difficile* spores	10^5^–10^7^ CFU/mL on agar plates and suspensions	UV-C (254 nm), UV-C lamp, fixed, wired; bench-top device	~2208 (for sporicidal effect) up to 30 min	Complete inactivation after 20 min at high dose; spores highly resistant	Not reported	[38]
*Candida albicans* biofilm	Inoculum ~2.39 × 10^6^ CFU/mL Biofilm formed on PMMA (polymethyl methacrylate) (denture base)	UV-C Hand-held UV-C lamp; wired, portable but not wireless	NA (lamp-based)	Reduced from 2.39 × 10^6^ to 2.15 × 10^5^ CFU/mL (~91% kill)	Not reported	[39]
*Pseudomonas aeruginosa*	Biofilms grown in CDC reactor Biofilms on Acrylonitrile Butadiene styrene(ABS- thermoplastic polymer), High-Density Polyethylene –(HDPE), Polycarbonate (PC), steel, silicone	UV-LED 280 nm, doses varied by surface wired; not mobile	NA (surface-dependent)	Log reductions depended on material: steel/PC → higher kill; silicone/HDPE → more resistant	Not reported	[40]

## Data Availability

The original contributions presented in this study are included in the article/Supplementary Materials. Further inquiries can be directed to the corresponding author.

## Supplementary Materials

The following supporting information can be downloaded at https://www.mdpi.com/article/10.3390/antibiotics15050507/s1, Table S1: Overall system specifications of the UVGI disinfection system; Table S2: CFU count for *B. subtilis*, *E. coli* K12, and *C. albicans* M-207 at different time intervals and distances and the corresponding UV dose; detailed operational steps and safety specifications are provided in the Supplementary Information.
