# Supplementary material for: Design of a Wireless Ultraviolet Germicidal Irradiation System and Validation of Germicidal Potential Against Biofilm-Forming Bacteria and Fungi
Source: Antibiotics (Basel). 2026 May 18;15(5):507. doi: 10.3390/antibiotics15050507 (PMC13203153; doi:10.3390/antibiotics15050507)
Supplement: Supplementary file 1 [file antibiotics-15-00507-s001.zip › antibiotics-4272134-supplementary (1).pdf]

Supplementary Data

Table S1. Overall system specifications of the UVGI Disinfection System.

Sl. No.	Parameters	Specification
1	Total UV Output	120 Watts
2	UV Tubes	8
3	Operation	Wi-Fi Control
4	Connectivity	> 20m Wireless
5	Battery Backup	3 h
6	Power Consumption	400 Watts
7	Individual Lamp Life	9000 h
8	Sterilization Time to Kill Microorganisms	10x10 Feet Room in 15 min
9	Coverage	360 Degree
10	Dimension	0.5x0.6x1.6 m
11	Weight	25 kg

Table S2. CFU count for *B. subtilis, E. coli* K12, and *C. albicans* M-207 at different time intervals and distances and the corresponding UV dose.

Cultures	Time (min)	UV Dose ($mJcm^{-2}$)	No of colonies	$CFUmL^{-1}$	UV Dose ($mJcm^{-2}$)	No of colonies	$CFUmL^{-1}$	UV Dose ($mJcm^{-2}$)	No of colonies	$CFUmL^{-1}$
		1m				3m			5m	
B. subtilis	0	0	494	4940000	0	494	494000000	0	494	494000000
	15	600.3	0	0	160.2	15	15000000	73.8	75	75000000
	30	1200.6	0	0	320.6	2	2000000	147.6	4	4000000
	45	1800.9	0	0	480.6	0	0	221.4	2	2000000
	60	2401.2	0	0	640.8	0	0	295.2	1	1000000
	90	3601.8	0	0	961.2	0	0	442.8	0	0
E. coli K12	0	0	566	566000000	0	566	566000000	0	566	566000000
	15	576	0	0	159.3	11	11000000	72	239	239000000
	30	1152	0	0	318.6	2	2000000	144	220	220000000
	45	1728	0	0	477.9	2	2000000	216	144	144000000
	60	2304	0	0	637.2	1	1000000	288	99	99000000
	90	3456	0	0	955.8	0	0	432	55	55000000
C. albicans M-207	0	0	875	875000	0	874	875000	0	874	875000
	15	697.5	0	0	318.6	7	7000	78.3	736	736000

30	1395	0	0	318.6	1	1000	156.6	613	613000
45	2092.5	0	0	318.6	1	1000	234.9	538	538000
60	2790	0	0	318.6	1	1000	313.2	398	398000
90	4185	0	0	318.6	1	1000	469.8	330	330000

Section S1 Methodology details

1. Device Operation Detailed Procedure

- Plug the red wire into the red connector (positive) of the battery and the black wire into the black connector (negative); never exchange the positions of the wires.
- Switch on the battery.
- Connect the UVGI system to the power source and switch on.
- The WiFi network is opened on the mobile/laptop and connected to the IP address of the system.
- Copy the IP address of the network and paste it in the browser.
- The browser then opens the control page to switch the UV tubes on/off using the relays.
- The experiments were performed, the relays were manually switched off, and the system and battery switch were switched off.
- Remove one of the connectors, either positive/negative

2. Safety Paradigm

Despite its formidable disinfection power, UV-C radiation carries inherent risks to human health, necessitating stringent safety protocols because this system is efficient in UV-mediated breaking of the DNA/RNA of an organism. With the primary concern of the potential for skin and ocular damage upon direct exposure, meticulous attention must be paid to safety measures. The system should be handled by trained professionals, and safety gear such as polypropylene face masks, polyester suits, and gloves, which exhibit very low transmissivity properties, should be worn during usage.

3. Safety Protocol for Operating UVC Room Sterilizers

- Operation of UVC sterilizers requires strict adherence to safety protocols to protect personnel from harmful exposure to UVC radiation (200–280 nm)
- Before the operation, the designated area must be cleared of all occupants. Entry should be restricted with visible warning signs indicating UVC sterilization in progress. Operators must wear personal protective equipment (PPE), including UV-blocking goggles with optical density >5, nitrile gloves, face masks, and full-sleeved UV-resistant garments. A UV protective kit should always be available at the worksite.
- During operation, personnel must avoid direct line of sight with active lamps. Remote or wireless control systems should be used to power the unit on and off, minimizing manual intervention.

- For real-world UVGI applications, strict adherence to safety protocols and PPE use is essential to prevent accidental UV-C exposure, which can damage skin and eyes.

4. Safety Measures Incorporating Specific Personal Protective equipment (PPE) and Goggles Specification

- PPE Material Selection: When selecting personal protective equipment (PPE), prioritizing materials resistant to UV-C radiation is imperative. Typically made of UV-resistant, tightly woven fabrics such as polyester-cotton blends or specialized UV-blocking materials. Garments composed of UV-resistant fabrics offer enhanced protection against skin damage.

- Gloves: Nitrile or neoprene gloves are preferred, as they resist UV degradation better than latex and protect hands during handling of UV equipment or contaminated materials.

- Goggle Specification: UV-resistant goggles or face shields/helmets made from polycarbonate or acrylic materials that block UV-C wavelengths (<280 nm). These prevent photokeratitis ("welder's eye") and long-term retinal damage. Utilization of UV-blocking goggles featuring lenses engineered to effectively filter UV-C radiation is paramount. Goggles with a high Optical Density (OD) rating, preferably surpassing OD 5, ensure robust ocular protection by attenuating the UV-C wavelengths. This mitigates the risk of ocular injury during disinfection procedures.

- Footwear: Closed shoes with UV-resistant coatings or standard laboratory safety shoes, which protect against accidental UV reflection off floors.

- Augmented Skin Protection: To fortify skin defences against UV-C exposure, the application of barrier creams or lotions must be considered. Formulations designed to provide comprehensive protection against chemical exposure or contact dermatitis enhance skin resilience and minimize potential irritant effects.

- Exposure Mitigation: Vigilantly mitigate direct exposure risks by refraining from gazing directly at activated UV-C tube lights and ensuring the absence of personnel within the vicinity during disinfection.

- Ventilation vigilance: Implementing robust ventilation mechanisms within the disinfection area to mitigate the accumulation of ozone, a byproduct of UV-C irradiation, is renowned for its deleterious health effects when inhaled at elevated concentrations.

5. Safety Protocols during Accidental exposure:

- Operate UVGI systems only in enclosed or controlled environments with interlock mechanisms and warning indicators.

- Workers must never be directly exposed to UV-C lamps while operational.

- In case of accidental exposure:

- Eyes: Immediately close eyes and move away from the source. Seek medical attention if irritation or pain persists.
- Skin: Cover affected area, avoid further UV exposure, and apply soothing creams (e.g., aloe vera); medical consultation may be required.
- Regular UV irradiance monitoring and equipment maintenance ensure safe and consistent operation.

These measures ensure safe handling of UVGI systems while maintaining their efficacy for microbial control.
